# Periauricular Angiolipoma: A Case Report

**DOI:** 10.29252/wjps.10.3.134

**Published:** 2021-09

**Authors:** Gholamreza Motazedian, Ali Khojasteh, Fatemeh Salari, Mohammad Hossein Anbardar

**Affiliations:** 1 Department of Plastic and Reconstructive Surgery, Shiraz University of Medical Sciences, Shiraz, Iran; 2 School of Dentistry, Shiraz University of Medical Sciences, Shiraz, Iran; 3 Department of Pathology, Namazee Hospital, Shiraz University of Medical Sciences, Shiraz, Iran

**Keywords:** Angiolipoma, Periauricular region

## Abstract

Angiolipoma is an uncommon benign fatty tumor which is a variant of lipoma. Microscopic studies on angiolipoma show that it includes the mature lipocytes and blood vessels. Infiltrating angiolipoma is an un-capsulated angiolipoma and, due to penetration into the surrounding structures, complete excision of the tumor is difficult. According to previous studies, the relapse rate of the infiltrating angiolipoma after surgical intervention is 35-50%. Infiltrating angiolipoma is rarely seen in the head and neck region and often occurs in the trunk and limbs. In this study, we report a 10-year-old boy with periauricular infiltrating angiolipoma who underwent surgery. After surgery, the patient developed transient left frontal branch palsy, but recovery was excellent and after one year there is no relapse.

## INTRODUCTION

Lipomas are fatty tumors and 5-17% of these tumors are angiolipomas(1). Angiolipoma is a variant of lipoma that includes mature lipocyts and blood vessels(2-6). Infiltrating angiolipoma are often placed deeper than the non-infiltrating form(1, 7). Infiltrating angiolipoma usually occurs in the trunk and limbs and is rarely seen in the head and neck region. Location of the tumor is a major factor in determining its prognosis(1). We present a patient with infiltrating angiolipoma in the periauricular region that underwent surgery.

## CASE REPORT

**Fig. 1:**
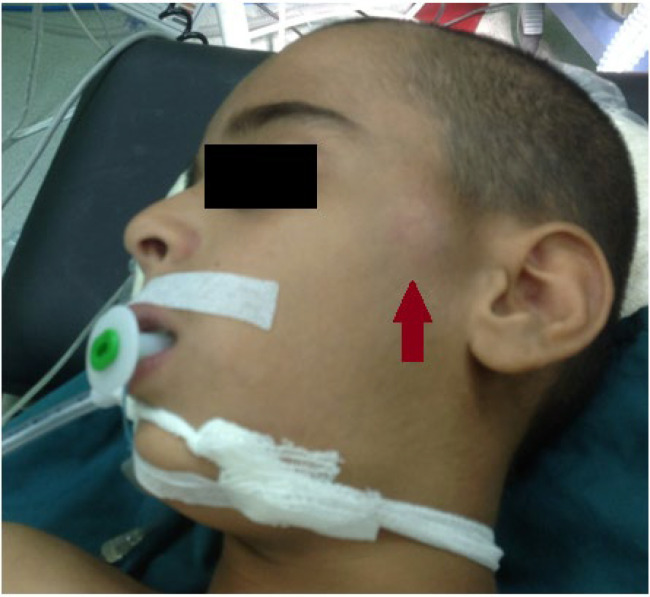
A 10-year-old boy with a mass on the left periauricular region

**Fig. 2:**
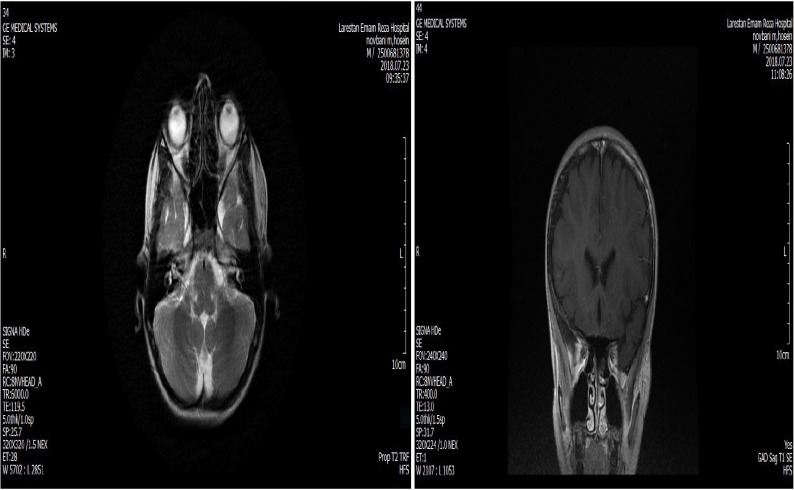
MRI revealed an ill-defined soft tissue mass

**Fig. 3:**
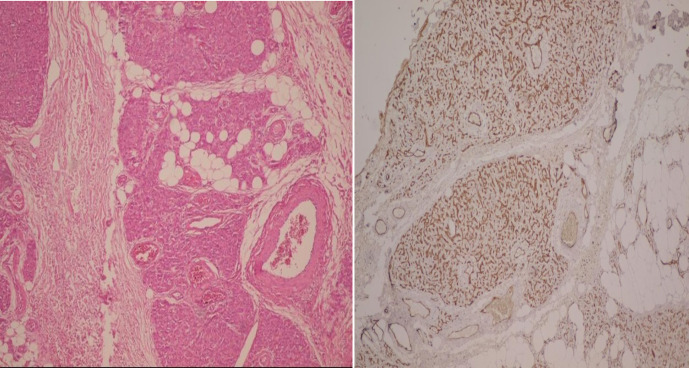
Section shows mature lipocyts and blood vessels indicating infiltrating angiolipoma

This study was conducted in August 2020 at Amiralmomenin hospital in Shiraz. A 10-year old boy presented with a mass on the left periauricular region with bluish discoloration during exercises (Figure 1). The patient had no history of trauma and infectious diseases. Physical exam showed a left periauricular painless subcutaneous mass. The facial magnetic resonance imaging (MRI) revealed ill-defined soft tissue mass in the left periauricular region. The tumor had hyper-intense signal on T2 weighted sequences and isointense signal on T1 weighted sequences (Figure 2). Fine needle aspiration was not diagnostic. Surgical excision of the un-capsulated mass was performed through periauricular incision under general anesthesia and loupe magnification. The specimen was sent for pathologic evaluation and infiltrating angiolipoma was reported (Figure 3). After surgery, the patient developed transient left frontal branch palsy, but recovery was excellent and after one year there is now no relapse.

## INFORMED CONSENT

Informed consent was obtained from the patient.(Ethical Code: IR.SUMS.REC.1400.405).

## DISCUSSION

Angiolipoma is a variant of lipoma that includes mature lipocyts and blood vessels(2-6). In different cases, the ratio of the lipocyt to the blood vessels varies, but in most of them the percentage of lipocyts is higher than blood vessels (4, 8). In terms of having or not having a capsule, they are divided into two categories: encapsulated (circumscribed) and un-capsulated (infiltrating) angiolipoma which penetrates the surrounding structures (9). Infiltrating angiolipoma usually occurs in the trunk and limbs and is rarely seen in the head and neck region. Location of the tumor is a major factor in determining its prognosis (7).

Angiolipomas usually happen sporadically, but a few cases have a family history of angiolipoma, and the inheritance pattern in these cases is autosomal recessive or in very rare cases autosomal-dominant pattern (5, 10). Also, in a study that examined the karyotype of three cases of angiolipoma, the researchers concluded that all three cases had abnormal karyotypes with loss or structural rearrangement of chromosome 13(8).

Histopathologic studies are required for definitive diagnosis of angiolipoma from other soft tissue tumors such as hemangioma, myxolipoma, and Kaposi’s sarcoma; magnetic resonance imaging (MRI) can be useful in evaluation of the tumor expansion and preoperative diagnosis (5, 11). Surgical excision is the best treatment for both encapsulated and un-capsulated angiolipomas (2, 3, 7). Because infiltrating angiolipoma often penetrates the surrounding tissues, complete resection of the tumor may damage the surrounding structures (5, 7). In the maxillofacial region, due to the existence of many vital structures, magnified operation is necessary to prevent damage to these structures, but in some situations sacrificing a branch of nerves is inevitable (12). According to previous studies, the relapse rate of infiltrating angiolipoma is 35-50%(6). Two possible causes of the recurrence of infiltrating angiolipoma after surgery are incorrect evaluation of the extent of the tumor and inadequate tumor resection during surgery (5).

## CONCLUSION

Angiolipoma is a variant of lipoma considered as one of differential diagnoses of the head and neck mass for proper treatment.

## CONFLICT OF INTEREST

The authors declare no conflict of interest.
